# A mid-Cretaceous enantiornithine foot and tail feather preserved in Burmese amber

**DOI:** 10.1038/s41598-019-51929-9

**Published:** 2019-10-29

**Authors:** Lida Xing, Ryan C. McKellar, Jingmai K. O’Connor, Kecheng Niu, Huijuan Mai

**Affiliations:** 1 State Key Laboratory of Biogeology and Environmental Geology, China University of Geosciences, Beijing, 100083 China; 2 School of the Earth Sciences and Resources, China University of Geosciences, Beijing, 100083 China; 3 Yingliang Stone Nature History Museum, Nan’an, 362300 China; 4 Royal Saskatchewan Museum, Regina, Saskatchewan S4P 4W7 Canada; 5 Biology Department, University of Regina, Regina, Saskatchewan S4S 0A2 Canada; 6 Department of Ecology & Evolutionary Biology, University of Kansas, 1501 Crestline Drive – Suite 140, Lawrence, Kansas 66045 USA; 7 Key Laboratory of Vertebrate Evolution and Human Origins of the Chinese Academy of Sciences, Institute of Vertebrate Paleontology and Paleoanthropology, Beijing, 100044 China; 8 Yunnan Key Laboratory for Palaeobiology, Yunnan University, Kunming, Yunnan 650091 China; 9 MEC International Laboratory for Palaeobiology and Palaeoenvironment, Yunnan University, Kunming, Yunnan 650091 China

**Keywords:** Palaeontology, Palaeoecology

## Abstract

Since the first skeletal remains of avians preserved in amber were described in 2016, new avian remains trapped in Cretaceous-age Burmese amber continue to be uncovered, revealing a diversity of skeletal and feather morphologies observed nowhere else in the Mesozoic fossil record. Here we describe a foot with digital proportions unlike any previously described enantiornithine or Mesozoic bird. No bones are preserved in the new specimen but the outline of the foot is recorded in a detailed skin surface, which is surrounded by feather inclusions including a partial rachis-dominated feather. Pedal proportions and plumage support identification as an enantiornithine, but unlike previous discoveries the toes are stout with transversely elongated digital pads, and the outer toe appears strongly thickened relative to the inner two digits. The new specimen increases the known diversity and morphological disparity among the Enantiornithes, hinting at a wider range of habitats and behaviours. It also suggests that the Burmese amber avifauna was distinct from other Mesozoic assemblages, with amber entrapment including representatives from unusual small forms.

## Introduction

The discovery of skeletal remains preserved in Cretaceous-age amber is one of the most intriguing developments in 21^st^ century palaeontology, with current research barely scratching the surface of the potential of these discoveries to shed light on the biology of long extinct organisms. To date, all diagnostic avian remains recovered from Burmese amber are referable to the Enantiornithes, the dominant clade of land birds in the Cretaceous. Recent work on avian material preserved in amber from Myanmar includes a series of studies on skeletal material with directly associated feathers^1–4^, parasites and pigments associated with isolated feathers that may belong Enantiornithes^5,6^, and unusual feather morphotypes that likely belong to the group^7^. Recovered specimens have been predominantly biased toward juvenile remains, providing the greatest evidence regarding details of precocial plumage in at least some members of this lineage. Most of the feathers described have been comparable to those found in modern birds in terms of the feather types and tracts represented, but they have not yet developed some derived features found in crown group avians. Enantiornithines in amber indicate that flight feathers in these stem birds had rachises that were less rigid (i.e., nearly cylindrical), and barbules that were poorly developed (i.e., hooklets connecting barbules are minimal or absent) e.g.^1,2^, as compared to modern birds. These specimens have also permitted the detailed study of two feather types associated with the Enantiornithes, rachis-dominated feathers, and scutellate scale filaments.

Biostratigraphic evidence suggests that Burmese amber dates to the middle–upper Albian (based on ammonites^8^), Albian–Cenomanian (based on palynology^9,10^), or Cenomanian–Turonian (based on arthropods^11^). Radiometric dating using U-Pb of zircons from the volcaniclastic matrix surrounding the amber has refined this age, providing an absolute estimate of 98.8 ± 0.6 Ma^12^. Here we describe amber inclusions consisting of a foot of an avian theropod, and a range of feathers representing multiple morphotypes. These inclusions are preserved in a single piece of amber from the Angbamo site, Tanai Township, Myitkyina District, Kachin Province of northern Burma (Myanmar). The amber piece is catalogued as specimen number YLSNHM01001, and measures 25.6 × 18.2 × 9.2 mm, with a weight of 2.68 g. The original specimen is housed in the Yingliang Stone Nature History Museum (=YLSNHM abbreviation) in Nan’an, China. The foot reveals a morphology previously unrecognized in Mesozoic avians, greatly increasing the functional diversity of Mesozoic birds. We describe the remains, which we argue are best referred to Enantiornithes, and explore possible functions for the unusual morphology observed.

## Results

### General morphology

**Figure 1:**
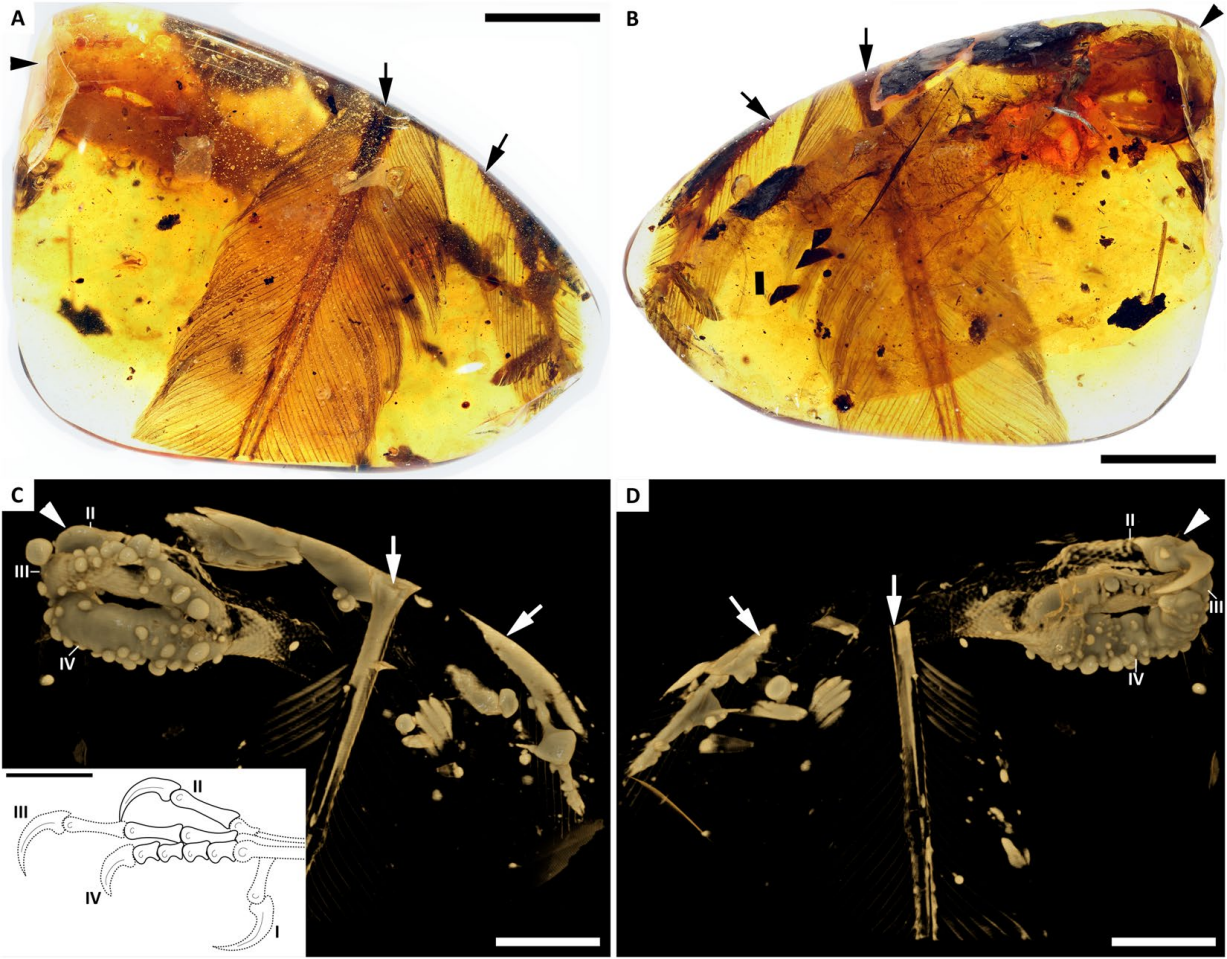
Overview and X-ray micro-CT renderings of YLSNHM01001. **(A,B)** Foot and flight feathers in dorsal and ventral views, respectively; with foot located ventral to feathers. **(C,D)** Corresponding micro-CT renderings with inset showing approximate reconstruction of digit proportions (additional details presented in Fig. 2). Arrowheads point to distal end of foot, inclined arrows to base of rectrix; vertical arrows to base of RDF; roman numerals indicate digit numbers. Scale bars = 5 mm.

**Figure 2:**
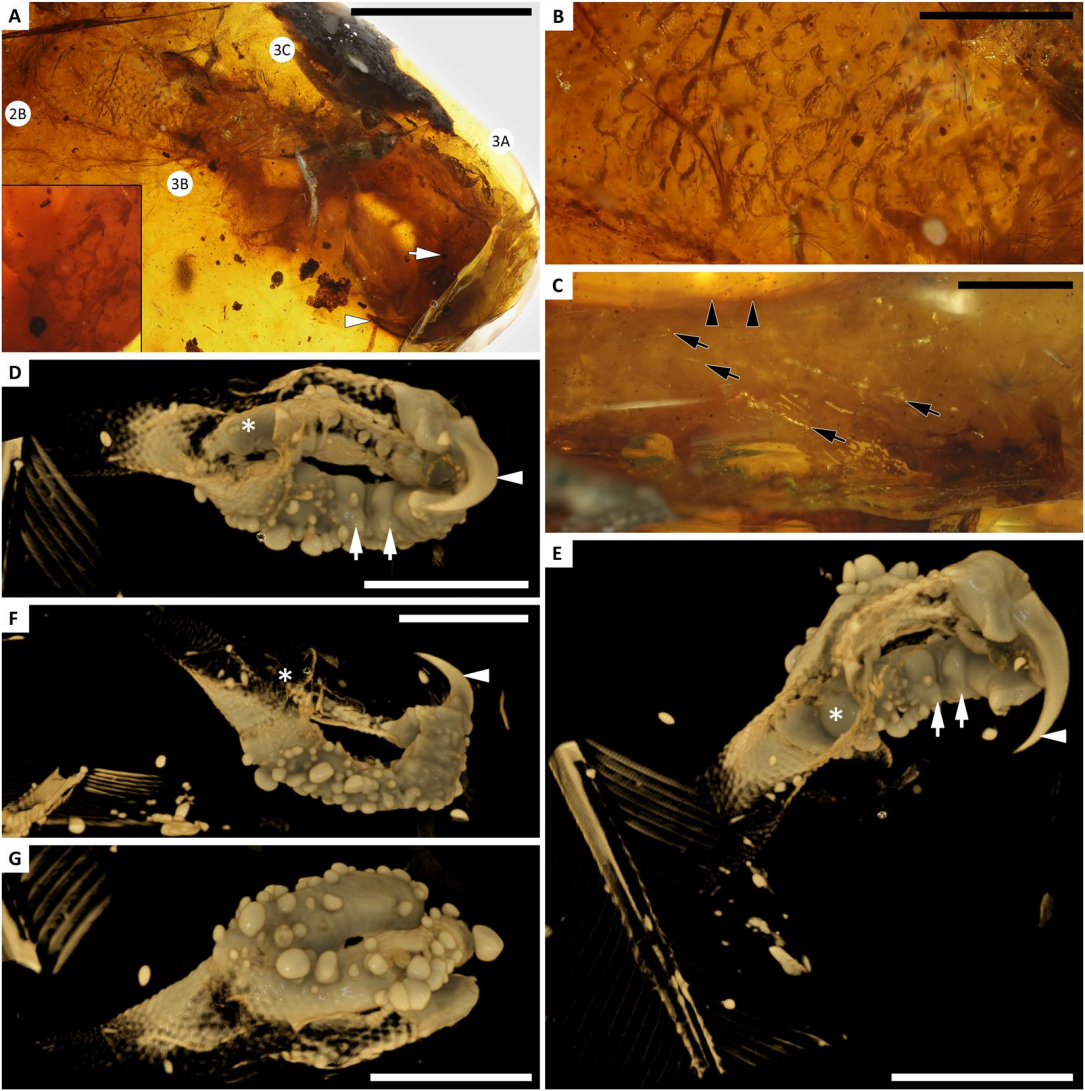
Detail of YLSNHM01001 foot inclusion. **(A)** Ventral view of foot, with arrowhead indicating ungual sheath, circled numbers indicating positions of higher magnification views in Figs 2 and 3, and inset showing reticulae on digital pad (near arrow). **(B)** Skin over metatarsals, with pervasive scutellae. **(C)** Dorsal surface of digit II, showing pale SSFs (arrows) originating from distal margins of scutellae (arrowheads). **(D–G)** External surface of foot; in predominantly ventral, medial, lateral, and dorsal views, respectively; showing taphonomic bubbles, broad transverse digital pads on digit IV (arrows), well-preserved ungual sheath on digit II (arrowheads), and surface breach of digit I (asterisks). Scale bars = 5 mm in A, D–G; 1 mm in B,C.

Although no bone is preserved, the integumentary surface of the foot permits some basic observations of digit proportions and outlines (Figs 1 and 2). The preserved surface demarcates the morphology of digits II–IV not including the unguals, belonging to a left foot. Only the keratinous sheath of digit II is preserved, whereas the ungual morphology of digits III and IV is ambiguous (Figs 1D and 2A,D–F). Based on the inferred position of the tarsometatarsal trochlea (established from the well-preserved skin demarcating the proximal portions of digits III and IV), the proximal phalanx in digit II was proportionately short and the trochlea of metatarsal II appears to have been in a more dorsal position relative to the trochlea of metatarsals III and IV. The trochlea of metatarsal III also appears to have extended slightly farther (distally) than that of metatarsal IV. We estimate the proximal phalanx of digit II was roughly half the length of the penultimate phalanx. The horny sheath of the digit II phalanx is subequal to the length of the penultimate phalanx. The ungual sheath is long, recurved, and laterally compressed, resembling those present in enantiornithines from the Jehol Lagerstätte e.g.^13^, as well as those in other enantiornithines from Hukawng^2,4^. Digit III was probably the longest digit, but closely followed by digit IV; however, without the unguals preserved, this interpretation is equivocal. The proximal two phalanges in digit III appear to be roughly subequal in length; the skin surrounding the penultimate phalanx is damaged and the length of this phalanx is impossible to accurately infer. Digit III appears more robust than digit II. What is most unusual about YLSNHM01001 is that digit IV appears to be more robust than digits II and III. The mediolateral thickness of digit IV is greater than that of either II or III (Figs 1C,D and 2D,G), whereas among Cretaceous birds (with the exception of hesperornithiforms), digits II or III are usually the most robust digits in the foot in both skeletal material and trackways. Typically digit IV is the most delicate and digit II is the most robust (e.g., *Eopengornis, Sulcavis*)^13,14^. The first phalanx appears to be the longest, followed by three shorter and subequal phalanges, as observed in numerous Jehol enantiornithines and stemward avians from the Jehol (e.g., *Sapeornis*, *Confuciusornis*). Although details of the hallux have been lost, and the relative widths of the toes may be influenced by taphonomy (discussed below), the overall morphology of the foot and the curvature of the preserved ungual sheath strongly suggest an arboreal bird^15–17^.

### Plumage and integument

**Figure 3:**
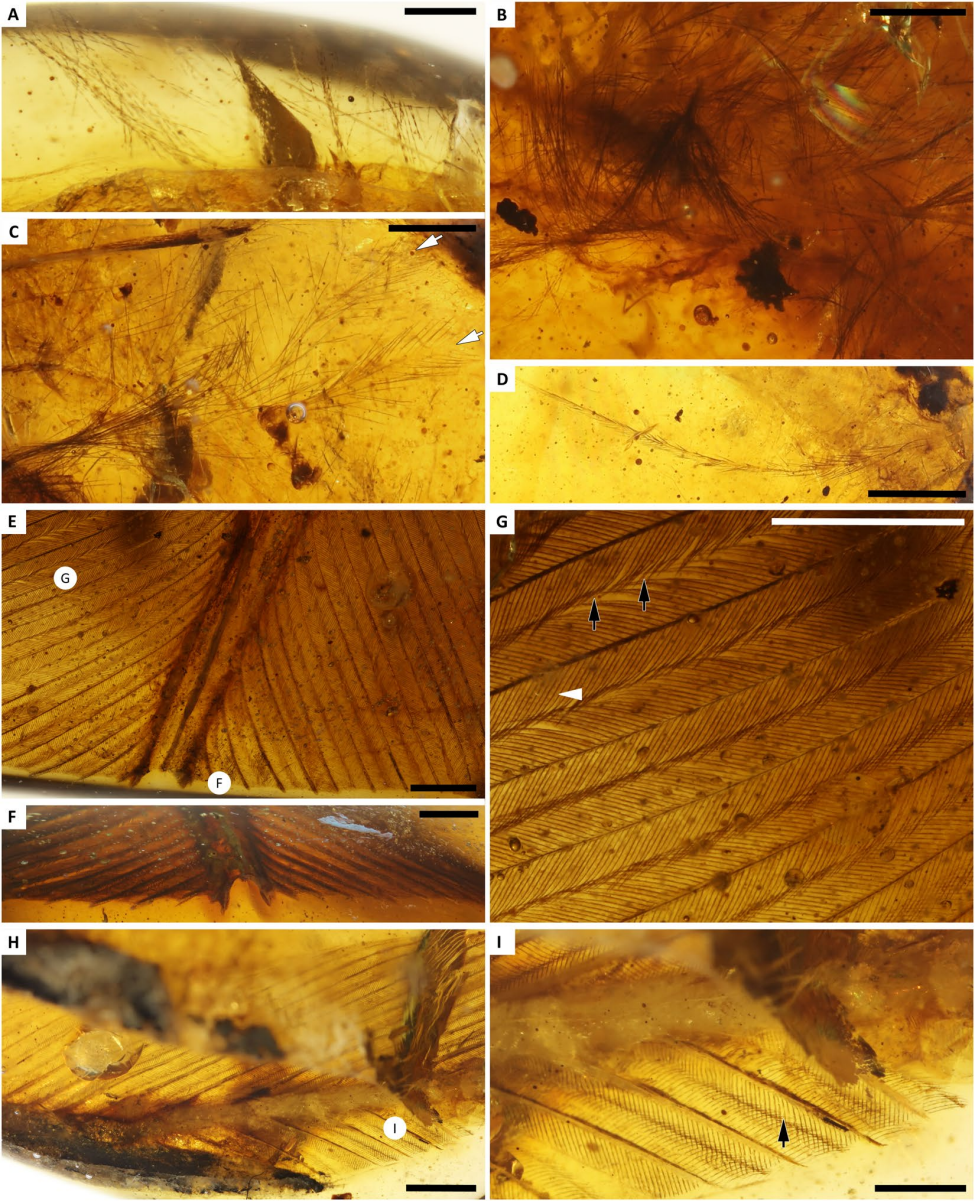
Detail of YLSNHM01001 feather inclusions. **(A)** Plumulaceous feather fragments in same flow as foot, with pigmentation concentrated in internodes of barbules. (**B**) Plumulaceous feathers in flow overlying foot, with diffuse brown pigmentation. (**C**) Contour feathers in overlying resin flow, that are plumulaceous proximally, pennaceous distally, with pale barb centers. (**D**) Isolated contour feather barb located near rectrix. (**E–G**) RDF in dorsal, cross-sectional, and magnified views, respectively; circled letters indicate position of images, opening in rachis faces ventrally, arrows indicate barbules with weak hooklets on pennulum, and arrowhead indicates barbule with scalloped pigmentation. (**H,I**) putative rectrix in ventral and magnified views, respectively; arrow indicates weakly developed pennulum. Scale bars = 0.25 mm in A; 0.5 mm in B,C,I; 1 mm in D–H.

The skin of the foot bears a range of feathers, and the surrounding amber captures small sections of feathers from other body regions (Figs 2C and 3). Two large pennaceous feathers are among the most prominent of the feathers trapped in the resin flow surrounding the soft tissue traces of the foot. The first of these feathers is a rachis-dominated feather (RDF), while the other is most likely a rectrix. Fragments of plumulaceous and contour feathers from other body regions are also found *ex-situ* in a subsequent resin flow that sealed the exposed ends of the metatarsals and digit I after weathering had taken place.

Pedal plumage includes a sparse coat of contour-like feathers overarching the dorsal surface of digit IV; however, these feathers are only represented by barb fragments that cannot be traced back to their insertion points or rachises. They have blade-shaped barbules that are dark brown apically and pale brown basally, giving the barbs pale cores. The skin surface of the foot lacks scutes, but has a dense covering of small scutellae on the dorsal and lateral surfaces (Fig. 2A,B). Scutellae on digits III and IV are accentuated by large bubbles that center on some scutellae; bubbles are largest on the laterobasal part of digit IV (Fig. 2D–G). The bubbles give the foot a ‘warty’ appearance, but these are taphonomic artifacts produced by decay and not true features of the pedal integument (see below). The dorsal surface of digits II–IV have a sparse coat of scutellate scale filaments (SSFs, *sensu* Xing *et al*.^2^) that lack pigmentation but reach lengths equivalent to the width of the adjacent digit. The SSFs appear most dense and elongate on the basal portion of digit II (Fig. 2C); however, they are difficult to observe in the other digits, being obscured by extensive decay products in the surrounding amber. Where visible, the SSFs consistently originate from the apex of each scutella. The plantar surface of the foot is largely obscured by fractures in the surrounding amber and overlap between the digits. Small sections of the base and apex of digit II show diminutive, pebbly reticulae, and a relatively prominent digital pad at the base of the ungual (Fig. 2A). CT data show much broader digital pads on the phalanges of digit IV (Fig. 2D,E). The metatarsals have fine, ellipsoidal scutellae dorsally and laterally, with finer, rounded scutellae ventrally (which clearly extend onto the plantar surface of digit II near its base); reticulae on the ventral surfaces of digits II–IV appear to be consistent in size.

The plumulaceous feathers stranded in the drying line dorsal to the foot have pale, diffuse, brown pigmentation visible. However, a few of the plumulaceous feathers exhibit a medium-to-dark brown colour, with pigmentation concentrated within the internodes and clear nodes (Fig. 3A). The internodes of these barbules appear nearly cylindrical, and flaring apically, but they lack obvious nodal prongs or expansions. These feathers are only visible near the very margins of the amber piece, limiting observations.

Comparison with other RDFs in amber suggests that the RDF feather section preserved in YLSNHM01001 encompasses the base of the distally vaned portion of a racket plume (similar to DIP-V-16223: Fig. 4^7^). The barbs near the base of the feather undergo a rapid reduction in length that is suggestive of a barbless region just basal to the preserved region. Barbs on either side of the rachis are nearly symmetrical, but those closest to the foot inclusion are slightly shorter (~7.1 mm vs. ~7.6 mm) and more apically directed (~41-degrees vs. ~45-degrees divergence from rachis). The laterally expanded rachis is clearly sectioned at the polished surface of the amber piece. The rachis exhibits the C-shaped profile characteristic of RDFs, with a prominent and narrow rachidial ridge, and ventral margins that are expanded to double the thickness of the adjacent laminae (Figs 2E,F and 3E,F)^7^. The ventral opening in the rachis faces toward the foot inclusion. Barbs are attached to the lateral surface of the rachis, well-removed from the ventral opening in the shaft, and the barb rami are blade-shaped (broad dorsoventrally). Both the barb rami and the rachis have a diffuse medium brown colouration (Fig. 2G). The pennaceous barbules have a weakly developed pennulum on the distal barbules, and very fine hooklets visible in some places. Pigmentation is diffuse and pale brown in the barbules, and is slightly reduced near the nodes, giving the basal blade of the barbules a mottled appearance.

An isolated remex or rectrix diverges from the RDF at an angle of approximately 40° (Figs 1A,B and 3H,I). The orientation and position of the RDF and flight feather are consistent with both feathers being part of the tail plumage, but these feathers have drifted significantly from their original anatomical position, and identification as a rectrix as opposed to a remex is inconclusive. The putative rectrix has a nearly cylindrical rachis that is full of milky amber and pith tissue. The rachis is wider than the adjacent barbs; barb rami are deeply blade-shaped, with dorsoventral thicknesses measuring approximately half that of the rachis thickness. The ventral part of each ramus is weakly pigmented, yet the dorsal portions have a dark brown colour. Barbules on the rectrix are reduced and blade-like (narrower than in the RDF), with distal barbules that clearly angle adapically at the base of their weakly defined pennulum with no visible hooklets, and proximal barbules that are relatively straight.

### Taphonomy

Syninclusions within YLSNHM01001 consist of a partial cupressaceous plant bract, plant trichomes (stellate hairs), numerous particulates, insect frass pellets, and other indeterminate plant foliage fragments. The high density of particulates and plant material within the specimen suggests that the resin mass was formed close to the forest floor^18^.

The foot appears to have been embedded in resin while it was still moist—the surrounding amber has a thick veil of milky amber and large decay bubbles that emanate from the surface of the foot^19^. All available data suggest that the foot was stranded on a drying line within the amber. The metatarsal region and the hallux have been obliquely truncated at this drying line (cut off midway through the metatarsals and near the base of digit I). Exposure to weathering on the drying line was prolonged enough that most of the soft tissue and bone decayed before a subsequent flow could infill and preserve the skin surface. The apices of digits III and IV appear to have been in contact with the edge of the amber piece, and are highly oxidized, but it is unclear if this breach occurred early or late in the taphonomic process. Regardless, this oxidation surface interferes with CT scanning in the ungual regions of these digits, leaving the ungual sheath of digit II as the only well-preserved example in the specimen. In many places the pedal integument has decayed and fragmented into sheets. The surface of the skin has a strong ‘warty’ appearance in CT data renderings, due to many large decay bubbles stemming from the surface of the foot. These bubbles are larger than the scutellae and suggest that the foot underwent significant decay in the resin before the resin polymerized and the exposed parts of the foot were weathered away.

Subsequent resin remobilization has forced downy feathers and a few contour feathers toward the distal end of the foot. One complete contour feather (plumulaceous basally, pennaceous distally) has been driven into the void where the metatarsals once sat (Figs 2A and 3C). The RDF and flight feather within the amber piece also appear to have been pushed anteriorly (toward the foot), but these feathers have maintained their orientations parallel to the resin flow layers, with their ventral surfaces facing the foot inclusion. The fact that the RDF has rotated perpendicular to the long axis of the foot, and that the distal vaned region is adjacent to the foot, suggests substantial anterior drift within the resin, but in a single direction and without much disturbance due to turbulence.

## Discussion

The partial foot preserved in YLSNHM01001 reveals yet another unusual pedal morphology that is undocumented in the diversity of stem birds known from the Early Cretaceous Jehol Biota, the most diverse currently known Mesozoic avifauna, or elsewhere. This adds to growing evidence that suggests the Hukawng avifauna was unique compared to other Cretaceous faunas. This discovery adds to the ecological diversity of Mesozoic birds, and its unexpected morphology leads to questions about potential functions, due to the lack of similar structures in the huge diversity of extant birds. YLSNHM01001 preserves only the skin and one ungual sheath, lacking the skeletal elements typically used for taxonomic identification in fossils, and rendering it difficult to assess the phylogenetic position of the new specimen. However, both the morphology of the single preserved claw and the preserved feather morphotypes associated with the specimen are suggestive of an enantiornithine source^7^. This is also consistent with the fact that Enantiornithes is the only clade documented in the Hukawng avifauna thus far. Without better-preserved material, and in the absence of any known Mesozoic bird with similarly robust pedal digits, any identification is tenuous. Nevertheless, referral to Enantiornithes is the simplest explanation for the morphology of the one preserved ungual sheath and the proportions of the lengths of the phalanges in digit IV. This identification is further supported by the presence of an RDF in the surrounding amber and SSFs on the surface of the foot, as these two feather types have only been found together in the Enantiornithes.

The absence of skeletal material in YLSNHM01001 limits the possibility of establishing the specimen’s ontogenetic stage, and the available plumage does not help to constrain the age of the specimen. Flight feathers have been recovered from enantiornithine compression fossils in developmental stages as early as the embryo^20,21^, and in association with osteologically immature enantiornithine remains from Burmese amber^1–3^, while RDFs are visible in compression fossils of juveniles e.g.^21,22^. Thus, the precocial plumage of enantiornithines limits the utility of feather morphotypes to be used in inferring ontogeny in YLSNHM01001 and other specimens. At most, it can be said that the SSFs present on the foot occur in density and lengths greater than those observed in a hatchling from the same deposit (HPG-15–1^2^), but less than those observed in a larger foot also from Burmese amber (DIP-V-15105^4^). Whether these differences are a function of age, or interspecific variation remains unknown. SSFs are unknown in Mesozoic birds from other deposits but have been found on all enantiornithine feet described from Hukawng, and may have become more pronounced in older individuals, or be more developed in some species (e.g., DIP-V-15105^4^). Tarsal plumage that is superficially similar to SSFs has also been noted in hatchlings of modern birds and have been produced through developmental manipulation e.g.^2,23,24^; however, the homology of these integumentary structures has not yet been tested.

Rachis-dominated feathers have been documented as elongate ornamental feathers in compression fossils of Enantiornithes (Early Cretaceous Jehol Group in China and the Crato Formation in Brazil), Confuciusornithiformes (Jehol), and potentially also the non-avian Scansoriopterygidae (Late Jurassic Daohugou Formation)^13,15^. In Enantiornithes, these feathers are typically found as paired tail streamers or racket plumes that can reach lengths that exceed that of the body of the bird. Recently, a series of 31 Burmese amber pieces containing RDFs was described, including 10 specimens in which the RDFs occurred in pairs^7^. Although these feathers were not recovered in direct association with skeletal material, they were most parsimoniously attributed to enantiornithines in light of the fact only enantiornithine skeletal remains have been discovered in Burmese amber thus far, and the fact that, among taxa known to possess RDFs, only enantiornithines are documented outside deposits from northeastern China^13,21,25^. The new specimen described here provides additional supports for the referral of previously described RDFs in Burmese amber to the Enantiornithes.

Taphonomy and morphology are both potential explanations for the unusual widths of the digits observed in YLSNHM01001. If taphonomy was the controlling factor in the preserved digit widths, digit II may be narrower than digits III and IV simply because its tissues dried out and shriveled from exposure prior to resin polymerization, while toes that were buried deeper within the resin mass preserved more accurate soft tissue outlines. This interpretation is partially supported by the greater extent of milky amber and decay-related bubbles surrounding digits III and IV. In this scenario, digit IV appears wider than the other digits because it was last (and least) affected by exposure prior to resin polymerization. However, this would mean that the unusual breadth of digit IV is still a true feature of this bird and bird toes lack musculature, so such significant differences in width are unlikely to be produced through desiccation alone. Foot morphology in YLSNHM01001 differs from all previously observed specimens in Burmese amber^2,4^. The anterior-facing digits in this scenario are proportionally broader than any other specimens observed to date, and digit IV has unique broad (transversely elongated) digital pads that are not present on the other digits.

If the observed digit widths represent the true morphological condition of the bird in *vivo* (i.e., digit IV was broader than II or III in life), much of the structure that is preserved in the YLSNHM01001 skin surface would have to come from underlying bone structure. The strongest evidence for this interpretation comes from the joint widths within the digits. In all three toes preserved, the joints are the widest parts of the digits. The joints appear more prominent in relation to phalanx shaft thicknesses in digit II, as they should based on comparison with living birds. Furthermore, the differences between digit II and digit IV cannot be completely attributed to loss of soft tissue thickness—particularly since the joints have a reduced thickness of overlying soft tissue to begin with, and the scutellae appear to have consistent dimensions on digits II–IV. Given the taphonomic features observed, it appears as though YLSNHM01001 had wider toes than any previously observed enantiornithine in Burmese amber, and that it likely had a foot in which digit IV was wider than digits II or III. The difference in widths may not have been as prominent as preserved, but the relative width of digit IV distinguishes YLSNHM01001 from any known Cretaceous bird and suggests a unique ecology for this bird.

Pedal morphology and proportions are strongly correlated with ecology. All known enantiornithines for which there is pedal material preserved appear to be arboreal, but data is strongly biased toward a single ecosystem represented by the Jehol Biota e.g.^15–17,26^. Although there currently appears to be a division between the ecology of enantiornithines versus that of ornithuromorphs (the former being primarily arboreal and the latter being primarily terrestrial), this dichotomy will likely collapse with the discovery of more material from a greater stratigraphic sample set. Yet this collapse has not happened through the discovery of the Hukawng avifauna—all enantiornithines from this avifauna uphold the hypothesis that this clade was primarily arboreal. In this context, the unusually wide and padded plantar surface of digit IV in YLSNHM01001 may be indicative of a mixed or atypical ecological role for this enantiornithine. The prominent plantar pads and papillose plantar surface (created by domed reticulae) are related to gripping substrates and prey^27^. The combination of strongly padded, robust digits with elongate claws is most similar to extant raptorial birds^28^, which may suggest YLSNHM01001 was a small aerial insectivore. This is an interpretation that has also been suggested for bohaiornithid enantiornithines in which the second pedal digit is the most robust. Future studies may benefit from widespread comparisons to small insectivorous birds, such as raptors and owls, in Recent arboreal settings. The unusual morphology of YLSNHM01001 may add support to the idea that ‘raptorial’ enantiornithines, in parallel to their extant relatives, utilized a diversity of pedal morphologies reflecting differences in prey capture.

## Methods

### Micro-CT scanning and 3D reconstruction

YLSNHM01001 was scanned with an X-ray micro-CT: Xradia 520 Versa (Carl Zeiss X-ray Microscopy, Inc., Pleasanton, USA) at the Yunnan Key Laboratory for Palaeobiology, Yunnan University, Kunming, China. The entire piece was scanned with a beam strength of 50 kV/4w for 168 minutes. The spatial pixel size was 26.59 µm, 26.59 µm and 26.59 µm, respectively. A total of 1,014 radiographs were registered in the scan and saved as TIFF stacks for further processing. Based on the obtained image stacks, structures of the specimen were reconstructed and isolated using Amira 5.4 (Visage Imaging, San Diego, USA). The subsequent volume rendering and animations were performed using VG Studiomax 2.1 (Volume Graphics, Heidelberg, Germany). Final figures were prepared with Photoshop CS5 (Adobe, San Jose, USA) and Illustrator CS5 (Adobe, San Jose, USA).

It was not possible to conduct destructive sampling on YLSNHM01001, to create exposures for SEM or TEM analyses of pigmentation. Consequently, we have limited inferences about feather colours to the visible traces preserved in the amber, and the intensity of pigmentation. Other specimens from this deposit have been analyzed with SEM, and have shown that eumelanosomes are visible within the darkly pigmented areas of comparable samples^29^, but the presence and types of chemical pigments within each sample, and their taphonomic histories remain largely unknown^5^.

### Terminology and abbreviations

Terminology used herein follows Lucas and Stettenheim^30^ for integumentary structure morphology, and Dove^31^ for feather barbule structure and pigment distribution. The use of RDF and anatomical terms specific to this feather type have been discussed at length e.g.^4^, while SSF is a recently established term^2^ free from assumptions regarding homology with protofeathers (*sensu* Prum^32)^. Taphonomic terms specific to amber largely follow those of Martínez-Delclòs *et al*.^33^.

Institutional abbreviations for specimens used as comparative material include: DIP—Dexu Institute of Palaeontology, Chaozhou, China, HPG—Hupoge Amber Museum, Tengchong City Amber Association, China; YLSNHM—Yingliang Stone Nature History Museum, Nan’an, China.

## Data Availability

The specimen studied (YLSNHM01001) is deposited in the Yingliang Stone Nature History Museum in Nan’an, China.
